# Impacts of a School-Based Intervention That Incorporates Nutrition Education and a Supportive Healthy School Canteen Environment among Primary School Children in Malaysia

**DOI:** 10.3390/nu13051712

**Published:** 2021-05-18

**Authors:** Choon Huey Teo, Yit Siew Chin, Poh Ying Lim, Shahril Azian Haji Masrom, Zalilah Mohd Shariff

**Affiliations:** 1 Department of Nutrition, Faculty of Medicine & Health Sciences, Universiti Putra Malaysia, UPM Serdang 43400, Selangor, Malaysia; amandahuey2004@yahoo.com (C.H.T.); zalilahms@upm.edu.my (Z.M.S.); 2 Department of Nutrition, Batu Pahat District Health Office, Johor State Health Department, Ministry of Health, Jalan Mohd Khalid, Batu Pahat 83000, Johor, Malaysia; 3 Research Centre of Excellence, Nutrition and Non-Communicable Diseases, Faculty of Medicine & Health Sciences, Universiti Putra Malaysia, UPM Serdang 43400, Selangor, Malaysia; 4 Department of Community Health, Faculty of Medicine & Health Sciences, Universiti Putra Malaysia, UPM Serdang 43400, Selangor, Malaysia; pohying_my@upm.edu.my; 5 Department of District Health Office, Batu Pahat District Health Office, Johor State Health Department, Ministry of Health, Johor Bahru 80000, Johor, Malaysia; shahrilazianmph@yahoo.com

**Keywords:** children, school nutrition program, nutrition education, school canteen, eating behaviors, physical activity, cognitive performance, body weight status, overweight, obesity, malnutrition

## Abstract

In this study, a school nutrition program (SNP) that incorporates nutrition education and a healthy school canteen environment was developed to improve nutrition knowledge among intervention respondents and provide a healthier environment for them to practice healthy eating. In the current study, we evaluated the impacts of the SNP on eating behaviors, physical activity, body mass index-for-age (BAZ), and cognitive performance at pre-intervention, post-intervention, and 3-month follow-up points between intervention and comparison groups. This intervention study involved 523 primary school children (7–11 years old) from six selected schools in Batu Pahat District, Malaysia. Each respondent completed anthropometric and cognitive performance assessments and a set of standardized questionnaire at pre-intervention, post-intervention, and 3-month follow-up points. Multiple linear mixed model analysis was performed to determine the impacts of that SNP after being adjusted for covariates. After the program, the intervention group increased their frequency of breakfast, lunch, and dinner consumption and morning tea snacking and showed more frequent physical activity and better cognitive performance as compared to the comparison group overtime (*p* < 0.05). At 3-month follow-up, the intervention group showed lower BAZ scores than their comparison counterparts (*p* < 0.05). The SNP showed positive effects on eating behaviors, physical activity, BAZ, and cognitive performance in school children. Hence, the SNP is highly recommended for all primary school children.

## 1. Introduction

Childhood overweight and obesity are increasing rapidly in both developed and developing countries, which is a major public health concern to many health authorities [1]. The South East Asia Nutrition Survey of Malaysian Children (SEANUTS Malaysia) reported that the prevalence of overnutrition was 21.6% among children aged 7–12 years in year 2013, which was higher than the prevalence of undernutrition 13.8% [2]. The recent National Health Morbidity Survey (NHMS) 2019 reported that the prevalence of overnutrition had increased to 29.8%, while the prevalence of undernutrition was 22.7% among children aged 5–17 years in the year 2019 [3]. The double burden of malnutrition problems in Malaysia [2,3,4,5,6] may affect the growth and development of children in later life. Children with obesity problems were shown to have higher risk of high blood pressure, high cholesterol [7], and impaired glucose tolerance in their adulthood [8], while children who are undernourished may have an increased risk of poor cognitive development and physical impairment [9,10,11]. Hence, action should be taken to combat the double burden of malnutrition in Malaysia, especially overweight and obesity problems among children.

The World Health Organization (WHO) has shown that changes in eating behaviors and physical activity pattern are the key factors in the high prevalence of childhood obesity [12]. Studies have also shown that school-aged children are at risk of poor dietary behaviors (such as skipping breakfast, low intake of fruits and vegetables, and unhealthy snacking) and low physical activity in Malaysia [13,14,15,16], which may affect their cognitive performance and nutritional status [11,17,18]. Based on the Malaysian National Health and Morbidity Survey (2017), only 33.1% of primary school children consume breakfast every day of the week. The reasons given by breakfast skippers were no appetite and no time in the early morning. In addition, approximately 43.8% and 52.9% of primary school children respectively had their lunch and dinner every day, with reasons for skipping being no appetite and on diet or effort to control body weight. Additionally, obese children and adolescents had the lowest prevalence rates of daily breakfast (26.2%), lunch (43.3%), and dinner (49.5%) consumption compared to their counterparts in other BMI categories [13]. In addition, the increasing prevalence of childhood overweight and obesity is related to both home and school canteen environments [19,20,21]. Unlike most adults, it is challenging for children to choose the environment where they live in or the foods they eat [22]. Therefore, it is imperative to plan comprehensive nutrition intervention programs that educate primary school children to practice healthy eating and active living in order to achieve good nutritional status and better cognitive performance.

Schools play important roles in combating the obesity epidemic, as children spend a significant amount of time in schools—six or more hours per day, 190 days per year, for six school years [23]. The school environment remains one of the best settings for school children to obtain their knowledge and skills regarding nutrition. Promoting healthy school nutrition is worth the effort, as it allows students to practice healthier behaviors, achieve better academic performance, and results in less school absenteeism [24,25]. It is beneficial to provide healthy eating knowledge and practice during childhood, since eating patterns are established in the early life and are difficult to change during youth [26]. Indeed, it is important to educate children about healthy eating and active living in the school setting, as it encourages them to practice healthy eating and active living during their school days, preventing them from experiencing overweight or obesity problems.

A systematic review reported that interventions focused on nutrition and health education are effective in increasing knowledge, attitudes, and practices regarding nutrition among adolescents [27]. Nutrition education interventions that focused on improving nutrition knowledge, attitudes, and practices (K.A.P) and reducing overweight and obesity rates among primary school children have been carried out in Malaysia [20,28,29]. The interventions were mainly focused on educating the children about healthy eating and physical activity. The interventions showed improvements in knowledge and attitudes regarding nutrition at post-intervention and 6-month follow-up, but no significant improvement in nutrition practices [28]. In order to improve nutrition practices, Tee et al. [3] suggested that an effective nutrition intervention should consist of content and teaching approaches that are developmentally applicable for the children and that tackle the changes in the school environment. To date, there is no existing intervention program that incorporates both nutrition education and a supportive healthy school canteen environment in promoting school nutrition among Malaysian children [30]. The food available in the school canteen must reflect what has been taught in the nutrition education, which can exert a strong influence on children’s food decisions [19,30,31]. Therefore, the present study proposed a school nutrition program (SNP) that comprised two main components, namely (i) nutrition education and (ii) a healthy school canteen environment [30]. This paper aims to report the effectiveness of the SNP on the eating behavior, physical activity, body composition, and cognitive performance among Malaysian primary school children.

## 2. Materials and Methods

### 2.1. Study Design and Participants

The study protocol for the SNP was published previously [30]. The study was a quasi-experimental study, whereby six primary schools were selected in Batu Pahat District, State of Johor, Malaysia. Out of 147 National Primary Schools in Batu Pahat district, a total of 143 National Primary Schools met the inclusion criteria of being co-educational, non-religious, non-special-educational, multi-ethnic, and government-funded. Next, the schools were stratified by school types, namely (i) National Primary School *(Sekolah Kebangsaan, SK)* (*n* = 104), (ii) National Chinese Primary School *(Sekolah Jenis Kebangsaan Cina, SJKC)* (*n* = 36), and (iii) National Tamil Primary School *(Sekolah Jenis Kebangsaan Tamil, SJKT)* (*n* = 3) groups. Two schools from each stratified group (SK, SJKC, and SJKT) were selected. From each stratified group, one school was assigned as IG and another school was assigned as CG. Three schools were assigned as an intervention group (one National Primary School *(Sekolah Kebangsaan, SK)*, one National Chinese Primary School *(Sekolah Jenis Kebangsaan Cina, SJKC),* and one National Tamil Primary School *(Sekolah Jenis Kebangsaan Tamil, SJKT),* while another three schools were assigned as a comparison group (one *SK, SJKC,* and *SJKT*, respectively). School principals, teachers, and canteen food handlers from all selected schools were invited to participate in the present study. Children who were enrolled in Standards 1 to 5, Malaysian, and either male or female from six selected schools were invited to attend the briefing on the school nutrition program prior to giving study consent. Upon request from the Malaysia Ministry of Education (MOE), all Standard 6 children, who will be attending the National Primary School Evaluation Test, were omitted form this study.

The sample size calculation for primary school children in the SNP was based on Aday and Cornelius’s (2006) equation [32]. The mean difference in waist circumference between intervention and control groups after the intervention program from a current intervention study in Malaysia, which involved primary school children aged seven to 11 years, was taken into consideration to calculate the sample size [33]. After adjusting for dropout rate, the calculated sample size was 244 each for intervention and comparison groups, respectively. The total sample size for the SNP should be at least 488 primary school children from Standards 1 to 5 in Batu Pahat, assuming a power of 80% and a level of significance of 5%. The small effect size of 0.09 was obtained.

While both intervention and comparison groups received the standard Physical and Health curriculum, the intervention group (IG) were invited to participate in the SNP. All outcome measures were assessed at pre-intervention (prior to the program), post-intervention (one week after completing SNP), and three-month follow-up (three-month follow-up without intervention).

### 2.2. Description of School Nutrition Program Intervention

Based on the Sustainable Development Goal (SDG) Three on Good Health and Well-Being, it is crucial to recognize the importance of prevention and health promotion in order to reduce the prevalence of malnutrition and the burden of diseases [34]. The SNP was based on Social Cognitive Theory (SCT), which emphasizes the importance of social and environmental factors in determining psychosocial and behavioral risk factors of both undernutrition and overnutrition problems [35]. The school nutrition program (SNP), which incorporates nutrition education and a healthy school canteen environment, was established to improve nutrition knowledge and practices among primary school children from the intervention schools and to create an environment to practice healthy eating habits.

The first component of the SNP was to deliver nutrition education to the children. The nutrition education comprised 17 topics, which included four main aspects, namely health awareness, nutrition, physical activity, and food hygiene. The researchers conducted training of trainers (TOT) with the teachers before conducting the School Nutrition Campaigns. The TOT for teachers aimed to provide hands-on training to the teachers to carry out nutrition education for primary school children during the School Nutrition Campaigns to empower them to convey nutrition messages to the children. A total of 41 trained teachers from the three intervention schools conducted nutrition education through three School Nutrition Campaigns (6 h/month) monthly for three months. The School Nutrition Campaigns were conducted in the classroom setting by the trained teachers during weekends. Therefore, the campaigns would not interfere with their school routine and would not burden the teachers, who had classes to teach on weekdays. When attending the campaigns, each of the children received an activity book and education materials, including a WHO Growth Chart 2007, Malaysian Food Guide Pyramid card, skipping rope, and a stationery set. The details of the SNP were previously described in [30].

Besides the campaigns, the nutritionists trained the canteen food handlers to prepare a healthy menu for the school children based on a standardized module. The healthy canteen training provided the knowledge and skills to serve healthier food, educating the food handlers from the three intervention schools in healthy cooking methods, the fundamentals of preparing healthy food, recipe modification, menu planning, and healthy cooking techniques. All canteen food handlers also needed to complete the basic requirements from the MOE Malaysia, including attending a Food Handling Course and receiving an anti-typhoid vaccine [36]. Together with the nutritionists in the SNP, the trained canteen food handlers discussed and proposed a four week menu during the one day workshop. In order to ensure the canteen food handlers applied the knowledge and skills learned, nutritionists instructed the food handlers to prepare four dishes based on the given menus. The school authorities and children tasted the dishes, and their feedback and comments were recorded to further improve the menu.

During school recess, the healthy menu was served to the children from 10.00 a.m. to 10.30 a.m. As recommended in the Healthy School Canteen Guidelines, the recommended energy per serving for a healthy menu during school recess for primary school children om Standards 1–3 is 150 kcal and for Standards 4–5 is 250 kcal [36]. The healthy menu contained four main food groups according to the Malaysian Healthy Plate concept, namely grains, vegetables, fruit, and protein foods. The researchers and trained teachers monitored all of the menus during the intervention period. With the healthy menu served, the children would be able to practice healthy eating during school recess. Support from parents and guardians, particularly in acceptance of the healthy menu during school recess, is crucial [19]. Parents from the Parent Teacher Association (PTA) were invited to visit the school canteen during recess time and to monitor the program’s implementation.

### 2.3. Measures

#### 2.3.1. Sociodemographic Characteristics

In the current study, the sociodemographic backgrounds of the children were determined (through both intervention and comparison groups) at baseline (pre-intervention). Data on age, sex, date of birth, ethnicity, parental education level, and monthly household income were obtained from parents. The parent-administered and child-administered questionnaires were prepared in Malay, Chinese, and Tamil languages. The information sheet and assent form were distributed to the children, whereby researchers verbally explained the study protocol in detail to the students, who were asked to pass on the information sheet and consent form to their parents. Children from Standards 1 and 2 were interviewed one-to-one by researchers, while children from Standards 3 to 5 self-administered the questionnaire. A concise summary of the questionnaire was done by researchers through a briefing. The researchers assisted the children for item clarification. Based on data from the Department of Statistics Malaysia (2018), the monthly household income groups were classified as B40 (total income less than RM 3860), M40 (total income from RM 3860 to RM 8319), and T20 (total income more than RM 8319) (1 USD = approximately to RM 4.11 on 16 March 2021) [37].

#### 2.3.2. Eating Behavior

The children’s main meal consumption and snacking behavior were assessed based on the Eating Behavior Questionnaire (EBQ) [16]. The EBQ assessed the frequency of breakfast, lunch, and dinner consumption, as well as the frequency of morning tea, evening tea, and supper consumption. The children were required to report their meal consumption and snacking behavior based on number of days such meals were consumed in a week [17].

#### 2.3.3. Physical Activity

The children’s physical activity patterns were examined using the Physical Activity Questionnaire for Children (PAQ-C) [38,39]. The types of physical activities were adapted to suit the Malaysian context, whereby some of the irrelevant activities were removed, such as hockey, cross-country skiing, and ice skating [40]. The 9-item PAQ-C is a 7-day physical activity recall instrument, with each item scored on a 5-point scale; a score of 1 indicates low physical activity, whereas a score of 5 indicates high physical activity. The Cronbach’s alpha for the PAQ-C instrument was 0.89.

#### 2.3.4. Body Weight Status

Children’s body weight and height were obtained to calculate the body weight status (BMI-for-age). Children’s body weight was measured using a SECA weighing scale. Children’s height was measured using a SECA stadiometer. Body weight and height were used for BMI calculations using the BMI formula BMI = weight (kg)/height^2^ (m^2^) [41]. WHO AnthroPlus software version 1.0.3 was used to analyze the mean z-score of BMI-for-age (BAZ) for all children [42] and compared with the z-score tables of the WHO Growth Reference 2007 [41]. All the assessments were performed by qualified nutritionists complying with the International Society for the Advancement of Kinanthropometry (ISAK) procedures.

#### 2.3.5. Cognitive Performance

The cognitive performance of the children was assessed by the researchers using the Raven’s Colored Progressive Matrices (CPM) [43]. The CPM consists of 36 items, grouped into three sets of 12 items (sets A, AB, and B) from the standard matrices. The children took 15–30 min to answer all 36 items, which were presented on a colored background. One mark was given for a correct answer, so the raw scores for the CPM test ranged between 0 and 36, whereby higher scores indicated better cognitive performance.

### 2.4. Ethical Approval

The study protocol obtained its research ethics approval from the National Medical Research Registry (NMRR) and Medical Research Ethics Committee (MREC) of MOH, Malaysia (NMRR-17-1273-36187 IIR). Permission to conduct the study was obtained from MOE Malaysia, State Department of Education of Johor, District Education Centre of Batu Pahat, and selected schools before conducting the study. The study protocol was explained to the children verbally, while the parents received the written information sheet. Informed assent was obtained from the children, while signed written consent was obtained from their parents or guardians, school authorities (principal and teachers), and canteen food handlers prior to data collection.

### 2.5. Statistical Analysis

The aim of current study was to evaluate the effectiveness of the SNP among Malaysian primary school children. A total of 1019 primary school children were invited to participate in the present study (IG: 621 children; CG: 389 children). A total of 582 children (IG: 303 children; CG: 279 children) returned the parental consent forms and child assent forms. A modified intention-to-treat analysis was used [44], whereby the children who fulfilled the following criteria were included in the data analysis process:

(i) Children in the intervention group who had attended at least two campaigns in the SNP;
(ii) Children, regardless of the intervention groups, who had completed pre-intervention, post-intervention, and 3-month follow-up sessions.

In order to fulfill the criteria, missing data for 9 children at 3-month follow-up (1.7%) were treated using the last observation carried forward (LOCF) approach [32]. Data from 523 children (IG: 251; CG:272) were analyzed by researchers to evaluate the effectiveness of the intervention.

All statistical analyses were conducted using IBM Statistical Package for the Social Sciences version 24.0. A chi-square test was used to determine the association between two or more groups of two categorical variables. An independent samples t-test was used to compare the mean values of the continuous variables between the intervention and comparison groups and a paired-sample t-test was used to compare mean values at pre-intervention and post-intervention points within the intervention and comparison groups, respectively.

A multiple linear mixed model was performed to investigate differences in eating behaviors, physical activity, BAZ, and cognitive performance between groups (intervention and comparison group) over time, adjusted with covariates (children’s age, sex, parental education level, and school type). Interaction terms of time and group were included in the model. The differences in all continuous variables between intervention and comparison groups were determined at three time points (pre-intervention, post-intervention, and 3-month follow-up). The statistical significance was set at *p* < 0.05.

## 3. Results

### 3.1. Sociodemographic Characteristics, Eating Behaviors, Physical Activity, Body Weight Status, and Cognitive Performance of the Children at Pre-Intervention

Table 1 shows that majority of the children were Malay (49.7%), followed by Chinese (30.4%) and Indian (19.9%), with a mean age of 8.95 ± 1.36 years. At pre-intervention, the percentage of males was 51.4%, however the IG had significantly higher numbers of males than the CG (*p* = 0.015). Two-thirds of the fathers and mothers had attained secondary education, while the mean parental monthly income was RM 2964.31 ± 2425.46 per month. Half of the children (51.4%) were classified as moderately active. Approximately half (50.5%) of the children had poor cognitive performance (below average and borderline) and one-third of the children had average (36.1%) cognitive performance. The prevalence of thinness was 7.8%, whereas the prevalence rates of overweight and obesity were 13.4% and 16.6%, respectively (Table 1).

At pre-intervention, the frequency of dinner consumption was found to be significantly higher in IG than the CG (*p* = 0.037), whereas no differences were found in the mean numbers of consumption days for breakfast, lunch, morning tea, afternoon tea, and supper snacking between IG and CG (breakfast: *p* = 0.838; lunch: *p* = 0.742; morning tea: *p* = 0.478; afternoon tea: *p* = 0.334 and supper: *p* = 0.900). IG and CG showed no difference in mean physical activity score at pre-intervention (*p* = 0.412). Both groups showed no significant difference in mean body weight, height, or BAZ (body weight: *p* = 0.717; height: *p* = 0.943; BAZ: *p* = 0.926). The distribution of body weight status showed no differences between IG and CG at pre-intervention (body weight status: *p* = 0.909). As for the mean cognitive performance, both groups showed no significant difference at pre-intervention (*p* = 0.365). The distributions of cognitive performance levels between IG and CG were not significantly different at pre-intervention (*p* = 0.488).

### 3.2. The Effectiveness of the School Nutrition Program

#### 3.2.1. Eating Behaviors

##### Within-Group Differences

Table 2 shows the changes in eating behaviors within IG and CG from pre-intervention to post-intervention and from pre-intervention to 3-month follow-up by using a paired-sample t-test. The children in the IG significantly increased in mean numbers of days of breakfast consumption and morning tea snacking at 3-month follow-up (breakfast: mean change = 0.55, t = 2.52, *p* = 0.012; morning tea snacking: mean change = 1.08, t = 5.68, *p* < 0.001), whereas no significant change was detected in the consumption patterns for lunch or dinner. The mean numbers of days of breakfast, lunch, and dinner consumption and morning tea snacking significantly decreased among the children in the CG over time (breakfast: post-intervention: mean change = −0.60, t = −3.02, *p* = 0.003; 3-month follow-up: mean change = −0.63, t = −3.14, *p* = 0.002; lunch: post-intervention: mean change = −1.26, t = −7.15, *p* < 0.001; 3-month follow-up: mean change = −0.98, t = −5.50, *p* < 0.001; dinner: post-intervention: mean change = −0.82, t = −4.52, *p* < 0.001; 3-month follow-up: mean change = −0.76, t = 4.30, *p* < 0.001; morning tea snacking: post-intervention: mean change = −0.50, t = −2.54, *p* = 0.012; 3-month follow-up: mean change = −1.06, t = −2.03, *p* < 0.001). Children in the IG significantly decreased in the mean numbers of days of afternoon tea snacking and supper snacking at 3-month follow-up (afternoon tea snacking: mean change = −0.75, t = −3.38, *p* = 0.001; supper snacking: mean change = −0.36, t = −2.02, *p* = 0.045), whereas no significant change was observed at post-intervention. On the other hand, children in the CG significantly decreased in the mean afternoon tea frequency at post-intervention (mean change = −0.52, t = −2.72, *p* = 0.007), but no change was observed at 3-month follow-up. There was no significant difference in the mean number of days of supper snacking among the CG over time.

##### Between-Group Differences Adjusted with Covariates

Table 3 shows the between-groups differences over time after being adjusted with covariates (children’s age, sex, parental education level, and school type) using a multiple linear mixed model. The IG increased dinner consumption by 0.54 units on average as compared to the CG (adjusted beta (β), 95% confidence interval (CI), *p*-value) (β = 0.54, 95% CI: 0.13, 0.95, *p* = 0.010). There were no significant differences in breakfast and lunch consumption or morning tea, afternoon tea, and supper snacking between groups.

The frequency of breakfast, lunch, and dinner consumption and of morning tea and afternoon tea snacking significantly decreased post-intervention (breakfast: β = −0.60, 95% CI: −1.00, −0.20, *p* = 0.003; lunch: β = −1.26, 95% CI: −1.58, −0.93, *p* < 0.001; dinner: β = −0.82, 95% CI: −1.15, −0.48, *p* < 0.001; morning tea: β = −0.50, 95% CI: −0.90, −0.10, *p* = 0.015; afternoon tea: β = −0.52, 95% CI: −0.92, −0.12, *p* = 0.011) and at 3-month follow-up (breakfast: β = −0.71, 95% CI: −1.10, −0.33, *p* < 0.001; lunch: β = −1.05, 95% CI: −1.36, −0.73, *p* < 0.001; dinner: β = −0.86, 95% CI: −1.17, −0.54, *p* < 0.001; morning tea: β = −1.06, 95% CI: −1.44, −0.69, *p* < 0.001; afternoon tea: β = −0.40, 95% CI: −0.81, −0.002, *p* = 0.049) compared to pre-intervention.

The interaction between time and group showed that the frequency of breakfast and lunch consumption and morning tea snacking among children in the IG significantly increased post-intervention (breakfast: β = 1.20, 95% CI: 0.57, 1.83, *p* < 0.001; lunch: β = 1.28, 95% CI: 0.77, 1.79, *p* < 0.001; morning tea: β = 0.84, 95% CI: 0.20, 1.48, *p* = 0.010) and at 3-month follow-up (breakfast: β = 1.57, 95% CI: 0.95, 2.18, *p* < 0.001; lunch: β = 1.27, 95% CI: 0.76, 1.77, *p* < 0.001; morning tea: β = 2.31, 95% CI: 1.70, 2.91, *p* < 0.001) compared to CG at pre-intervention. The dinner consumption in the IG significantly increased at 3-month follow-up (β = 0.98, 95% CI: 0.48, 1.47, *p* < 0.001) but did not change post-intervention. There was no significant difference in afternoon tea or supper snacking between both groups after intervention (Table 3).

##### Covariate Effects

Table 3 shows the effects of covariates on eating behaviors as assessed using a multiple linear mixed model. When comparing the effect of the children’s age on eating behaviors, children aged 7–9 years increased their supper snacking compared to children aged 10–11 years old (β = 0.32, 95% CI: 0.03, 0.60, *p* = 0.030). There were no significant differences in breakfast, lunch, and dinner consumption or in morning tea and afternoon tea snacking between both age groups. Boys increased in supper snacking compared to girls (β = 0.33, 95% CI: 0.06, 0.60, *p* = 0.018). No significant differences in breakfast, lunch, and dinner consumption or in morning tea and afternoon tea snacking were observed between boys and girls.

When comparing the parental education level, children whose father attained tertiary education showed increased breakfast consumption compared to children whose father attained secondary education (β = 0.58, 95% CI: 0.03, 1.12, *p* = 0.039). On the other hand, children whose mother attained tertiary education decreased in breakfast consumption compared to children whose mother attained secondary education (β = −0.67, 95% CI: −1.18, −0.16, *p* = 0.011). There were no significant differences in lunch and dinner consumption or in morning tea, afternoon tea, and supper snacking based on parental education level.

When comparing the school types, children from SJKC showed increased breakfast and dinner consumption but reduced afternoon tea snacking as compared to their SK counterparts (breakfast: β = 1.32, 95% CI: 0.96, 1.68, *p* < 0.001; dinner: β = 2.19, 95% CI: 1.88, 2.50, *p* < 0.001; afternoon tea: β = −1.15, 95% CI: −1.52, −0.78, *p* < 0.001). There were no significant differences in lunch consumption or morning tea and supper snacking between SK and SJKC counterparts. Children from SJKT increased their breakfast, lunch, and dinner consumption and morning tea, afternoon tea, and supper snacking as compared to their SK counterparts (breakfast: β = 0.66, 95% CI: 0.24, 1.07, *p* = 0.002; lunch: β = 1.11, 95% CI: 0.14, 2.08, *p* = 0.025; dinner: β = 1.52, 95% CI: 1.17, 1.88, *p* < 0.001; morning tea: β = 0.96, 95% CI: 0.58, 1.34, *p* < 0.001; afternoon tea: β = 0.79, 95% CI: 0.37, 1.22, *p* < 0.001; supper: β = 1.37, 95% CI: 1.00, 1.74, *p* < 0.001).

#### 3.2.2. Physical Activity

##### Within-Group Differences

Table 2 shows that the children in the IG increased their mean physical activity scores post-intervention and at 3-month follow-up (post-intervention: mean change = 0.37, t = 7.12, *p* < 0.001; 3-month follow-up: mean change = 0.18, t = 3.26, *p* = 0.001), while no significant changes in mean physical activity were observed among children in the CG over time.

##### Between-Group Differences Adjusted with Covariates

Table 3 shows no significant differences based on group or time spent on physical activity. However, the physical activity among children in IG increased on average by 0.34 units post-intervention (β = 0.34, 95% CI: 0.20, 0.47, *p* < 0.001) and 0.19 units at 3-month follow-up (β = 0.19, 95% CI: 0.05, 0.33, *p* = 0.008) compared to CG at pre-intervention after adjustment with covariates.

##### Covariate Effects

Boys showed higher physical activity than girls by 0.14 units (β = 0.14, 95% CI: 0.07, 0.22, *p* < 0.001). Children from SJKC showed higher physical activity compared to their SK counterparts (β = 0.15, 95% CI: 0.06, 0.24, *p* = 0.001). There were no significant differences detected based on children’s age or parental education level (Table 3).

#### 3.2.3. Body Weight Status

##### Within-Group Differences

Table 2 shows that the mean body weight and height increased within both groups over time. However, the children in the CG showed greater increases in mean body weight and height (body weight: post-intervention: mean change = 1.59, t = 10.86, *p* < 0.001; 3-month follow-up: mean change = 3.11, t = 23.63, *p* < 0.001; height: post-intervention: mean change = 2.13, t = 20.37, *p* < 0.001; 3-month follow-up: mean change = 3.66, t = 23.70, *p* < 0.001)) than children in the IG (body weight: post-intervention: mean change = 1.15, t = 7.21, *p* < 0.001; 3-month follow-up: mean change = 2.37, t = 12.29, *p* < 0.001; height: post-intervention: mean change = 1.95, t = 24.84, *p* < 0.001; 3-month follow-up: mean change = 3.60, t = 31.15, *p* < 0.001). The mean BAZ among children in the CG increased post-intervention (mean change = 0.08, t = 2.15, *p* = 0.033) and at 3-month follow-up (mean change = 0.20, t = 5.83, *p* < 0.001) as compared to pre-intervention, while no significant changes were observed among children in the IG over time (Table 3).

##### Between-Group Differences Adjusted with Covariates

Table 4 shows no significant differences in body weight, height, or BAZ between IG and CG. The body weight, height, and BAZ showed significant increases at post-intervention and 3-month follow-up points as compared to pre-intervention (post-intervention: body weight: β = 1.59, 95% CI: 1.34, 1.83, *p* < 0.001; height: β = 2.13, 95% CI: 1.96, 2.31, *p* < 0.001; BAZ: β = 0.08, 95% CI: 0.02, 0.14, *p* = 0.011; 3-month follow-up: body weight: β = 3.00, 95% CI: 2.76, 3.23, *p* < 0.001; height: β = 3.55, 95% CI: 3.29, 3.81, *p* < 0.001; BAZ: β = 0.19, 95% CI: 0.13, 0.25, *p* < 0.001). However, the interaction between time and group showed that the children in the IG decreased body weight on average by 0.47 units and BAZ on average by 0.20 units at 3-month follow-up compared to CG at pre-intervention (body weight: β = −0.47, 95% CI: −0.85, −0.09, *p* = 0.016; BAZ: β = −0.20, 95% CI: −0.30, −0.11, *p* < 0.001); however, no differences were shown post-intervention.

##### Covariates Effect

Table 4 shows children aged 7–9 years showed lower body weight by 6.37 units and height by 12.08 units compared to children aged 10–11 years (body weight: β = −6.37, 95% CI: −7.89, −4.85, *p* < 0.001; height: β = −12.08, 95% CI: −13.52, −10.63, *p* < 0.001). Boys reported higher body weight and BAZ compared to girls (body weight: β = 1.96, 95% CI: 0.50, 3.42, *p* = 0.009; BAZ: β = 0.49, 95% CI: 0.18, 0.79, *p* = 0.002). Children from SJKC showed increased height by 4.07 units as compared to SK counterparts (height: β = 4.07, 95% CI: 2.44, 5.70, *p* < 0.001. Children from SJKT had lower body weight but higher height compared to SK counterparts (body weight: β = −2.96, 95% CI: −4.94, −0.99, *p* = 0.003; height: β = 2.70, 95% CI: 0.82, 4.58, *p* = 0.005). There were no significant differences in body weight, height, or BAZ based on parental education level.

#### 3.2.4. Cognitive Performance

##### Within-Group Differences

Both IG and CG increased in their mean cognitive performance scores (IG: post-intervention: mean change = 13.12, t = 14.29, *p* < 0.001; 3-month follow-up: mean change = 19.17, t = 18.15, *p* < 0.001; CG: post-intervention: mean change = 9.67, t = 11.35, *p* < 0.001; 3-month follow-up: mean change = 14.78, t = 14.80, *p* < 0.001). However, children in the IG showed greater increases as compared to children in the CG over time (Table 2).

##### Between-Group Differences Adjusted with Covariates

After adjustment with covariates, children showed increased cognitive performance by 9.67 units at post-intervention and 13.71 units at 3-month follow-up as compared to pre-intervention (post-intervention: β = 9.67, 95% CI: 7.97, 11.37, *p* < 0.001; 3-month follow-up: β = 13.71, 95% CI: 11.79, 15.63, *p* < 0.001). The interaction between time and group showed that cognitive performance among children in the IG increased on average by 3.98 units at post-intervention and 4.35 units at 3-month follow-up compared to the CG at pre-intervention (post-intervention: β = 3.98, 95% CI: 1.28, 6.69, *p* = 0.004; 3-month follow-up: β = 4.35, 95% CI: 1.29, 7.41, *p* = 0.005) (Table 4).

##### Covariates Effect

Children from SJKC showed higher cognitive performance (β = 5.94, 95% CI: 3.27, 8.61, *p* < 0.001) whereas children from SJKT showed lower cognitive performance as compared to SK counterparts (β = −8.00, 95% CI: −11.08, −4.93, *p* < 0.001). There were no significant differences in cognitive performance based on children’s age, sex, or parental education level (Table 4).

## 4. Discussion

The current study was conducted to evaluate the effectiveness of the school nutrition program (SNP), which combined both nutrition education and a healthy school canteen environment for primary school children. To the best of our knowledge, this is the first health-promoting school program that has combined nutrition education with a supportive, healthy school canteen environment among Malaysian primary school children. After the SNP, the intervention group (IG) increased the frequency of breakfast, lunch, and dinner consumption and morning tea snacking and showed higher physical activity and better cognitive performance as compared to the comparison group overtime after adjusting with covariates. At 3-month follow-up, the IG showed lower BAZ than their comparison counterparts after adjusting with covariates.

Children in the IG significantly increased in their frequency of breakfast and lunch consumption and of morning tea snacking as compared to CG at post-intervention and 3-month follow-up after adjusting with covariates. Moreover, the IG significantly increased in their frequency of dinner consumption at 3-month follow-up as compared to the CG. The SNP emphasized the importance of consuming meals on schedule, which taught the IG about ways to avoid unhealthy eating practices, such as skipping meals. Regular meal consumption, particularly breakfast consumption, has been associated with better overall food intake and better cognitive and academic performance in children and adolescents [14,45,46,47,48,49]. It is crucial to improve the eating behavior of children to prevent the future burden of non-communicable diseases [50,51,52,53].

In addition, the SNP promoted a healthy school canteen environment that served different types of healthy meals every school day during morning tea snacking period (school recess time), which is an important time to provide energy to help the children to continue their studies, to practice classroom nutrition education messages, to increase attendance, or to decrease absenteeism. The healthy canteen significant reduced the availability of processed food, high energy density food, and sugary drinks to primary school children [19,25,31,54,55,56,57]. The school canteen from the intervention group served the healthy menu to the school children during the intervention period. At the same time, school children from the intervention group attended three School Nutrition Campaigns, which empowered them with nutrition knowledge and skills. The current results were consistent with a study in United States, in which the nutritional intake among elementary school children was strongly affected by the school canteen [19]. Given the positive environment’s influence on children’s food choices, it is crucial to create a healthy food environment in schools that helps primary school children to choose healthy food.

After the SNP, the children in the IG increased in physical activity at post-intervention and 3-month follow-up, which was consistent with past studies among primary school children [58,59]. In Malaysia, children and adolescents are suggested to perform moderate- or vigorous-intensity physical activity for at least 60 min daily [15]. The improvement in the physical activity in the SNP may be attributed to the various physical activities run by the trained teachers and the nutrition education, which emphasized the importance of physical activity and a reduction in sedentary activity.

In the present study, one in three primary school children was either overweight or obese, while one in ten was thin in Batu Pahat District, Malaysia. The finding from the present study was consistent with MyBreakfast study and NHMS (2019) in Malaysia [3]. The present results also demonstrated that the prevalence of obesity among IG reduced from 17.1% to 15.1% after 3-month follow-up, which is probably explained by the intentions of the children to control their weight using the knowledge and skills gained in the SNP and being supported by the healthy school canteen environment. In the present study, children in the IG showed significantly lower weight and BAZ compared to the CG at 3-month follow-up after adjusting with covariates. Consistent with two local obesity intervention studies [33,60], the BAZ was significantly lower in IG than CG after 3-month follow-up.

Almost half of the children (50.5%) showed poor cognitive performance (below average or borderline), which was higher than in a Southeast Asian study that involved 2127 Malaysian school-aged children [11]. In the present study, the children in the IG significantly improved their cognitive performance scores over time. The SNP increased the frequency of breakfast consumption and morning tea snacking among children in the IG, who tended to have a lower BAZ [61,62], which may improve cognitive performance, consistent with the studies by Pieper et al., Hoyland et al., and Li et al. [30,63,64]. On the other hand, another study demonstrated that dinner consumption was associated with cognitive performance, whereby children who consumed dinner at least five days a week were found to perform better on cognitive tasks as compared to those who had dinner less than five days a week [18]. Therefore, nutrition interventions among school-aged children are urgently needed, as these can maximize their optimal linear growth potential and may prevent them from experiencing malnutrition problems [65].

The CG in the current study, who received no intervention, tended to skip meals more frequently than the IG, which might have been due to time constraints, oversleeping in the morning, or a lack of appetite or stomach ache in the early morning [20,66,67]. Previous cross-sectional studies showed that skipping meals, especially skipping breakfast, was associated with higher body weight and BAZ and associated co-morbidity in children as compared to non-breakfast skippers [2,5,16,62,68,69,70], similar to the current results. Studies have also shown that a lack of consistent access to nutritious and safe food can have detrimental and long-lasting effects on the physical, cognitive, and psychosocial development of children [70]. Therefore, providing healthy meals in school canteens is crucial to improve primary school children’s eating behaviors, which are formed early in life and persist into adulthood.

The current study has several strengths. The uniqueness of the SNP is its incorporation of nutrition education and a healthy school canteen environment, an approach that improved healthy lifestyle behaviors, such as eating behaviors, physical activity, BAZ, and cognitive performance, among Malaysian primary school children in the Batu Pahat district. The involvement of the school principal, teachers, and canteen food handlers is important for SNP sustainability. The school principal included all of the TOTs as part of the teachers’ in-service training, while school canteen food handlers had to complete the training in order to renew their school canteen contracts. The trained teachers and canteen food handlers provided positive feedback about the training and found the content useful to them. Several studies that included Cochrane reviews reported that in interventions focusing on children (aged 6–12 years), those found to have significant impacts were those that included a school curriculum addressing healthy eating and physical activity and that improved the nutritional quality of foods offered at schools and of teacher involvement [19,71,72], many of which were included in the present study. In addition, the provision of healthy meals during recess was similar in a previous school meals program, which was associated with increased attendance and decreased absenteeism, a possible explanation for the improvement in academic performance [63,73]. In the current study, the majority of the children belonged to medium household income families; therefore, the cost of healthy meals for the children (RM 2 per meal) was considered as appropriate and cost-effective. A Cochrane review of school meal provision to disadvantaged children indicated some benefits in terms of physical and psychosocial health [74].

On the other hand, the limitation that should be considered in the present study was the use of data that were self-reported by children. The accuracy of the data was dependent on the honesty and ability of the children to recall their eating behaviors over the past week, which may cause self-reported bias and recall bias. Secondly, due to time, financial, and human resource constraints, the implementation of the SNP was limited to children from Batu Pahat District, State of Johor. Therefore, the impact of the SNP cannot be generalized to children from other districts with different socioeconomic backgrounds.

## 5. Conclusions

The three-month SNP incorporated nutrition education and a healthy school canteen environment, along with an another three months of follow-up. It was effective in improving eating behaviors, physical activity, and cognitive performance and in lowering BAZ among primary school children in Batu Pahat District, Malaysia. The concept, content, presentation, and support from the teachers and canteen food handlers were the major contributions to the success of the intervention. The SNP not only inspired the schools to change the offerings in the school canteens, but also supported the teachers to embed their knowledge of healthy nutrition in the curriculum and to develop healthy school food policies. Future studies may consider incorporating nutrition education and healthy school canteen environments while expanding the program to a bigger scale in Malaysia and evaluating the long-term impacts on children’s overall lifestyles. Policy makers, country leaders, program planners, and health agencies can use the data obtained from the present study to develop and implement effective policies that comprise nutrition education and healthy school canteen environments for Malaysian children in order to promote healthy lifestyles and reduce the incidence of malnutrition and the burden of disease among primary school children, which is suggested in the Sustainable Development Goals. 

## Figures and Tables

**Table 1:** Sociodemographic characteristics, eating behaviors, physical activity, body weight status, and cognitive performance of the children in intervention and comparison groups at pre-intervention (*n* = 523).

Characteristics	Respondents	Mean ± SD/n (%)		*t*-Test/Chi-Square, *p*-Value ^1^
	Total (*n* = 523)	Intervention (*n* = 251)	Comparison (*n* = 272)	
**Sociodemographic characteristics**				
Sex				χ^2^ = 5.93, *p* = 0.015 *
Boys	269 (51.4)	143 (57.0)	126 (46.3)	
Girls	254 (48.6)	108 (43.0)	146 (53.7)	
**Age (years)**	8.95 ± 1.36	8.90 ± 1.36	8.99 ± 1.36	t = −0.78, *p* = 0.438
**Ethnicity**				χ ^2^ = 2.15, *p* = 0.341
Malay	260 (49.7)	132 (52.6)	128 (47.1)	
Chinese	159 (30.4)	69 (27.5)	90 (33.1)	
Indian	104 (19.9)	50 (19.9)	54 (19.8)	
**Parental Monthly Income ^2^ (RM)**	2964.32 ± 2425.46	3156.31 ± 2514.85	2787.15 ± 2330.56	t = −0.66, *p* = 0.510
B40 (<RM3860)	399 (76.3)	181 (72.1)	218 (80.1)	χ^2^ = 0.25, *p* = 0.616
M40 (RM3860-8319)	104 (19.9)	56 (22.3)	48 (17.6)	
T20 (>RM8319)	20 (3.8)	14 (5.6)	6 (2.2)	
**Father’s Education Level**	Total (*n* = 449)	Intervention (*n* = 177)	Comparison (*n* = 272)	χ^2^ = 3.31, *p* = 0.191
Primary or below	56 (12.5)	22 (12.4)	34 (12.5)	
Secondary	333 (74.2)	125 (70.6)	208 (76.5)	
Tertiary	60 (13.4)	30 (16.9)	30 (11.0)	
**Mother’s Education Level**	Total (*n* = 449)	Intervention (*n* = 177)	Comparison (*n* = 272)	χ^2^ = 5.59, *p* = 0.061
Primary or below	68 (15.1)	30 (16.9)	38 (14.0)	
Secondary	309 (68.8)	111 (62.7)	198 (72.8)	
Tertiary	72 (16.0)	36 (20.3)	36 (13.2)	
**Main meal consumption** (Mean number of days in a week, 0–7)				
Breakfast	4.88 ± 2.67	4.90 ± 2.69	4.86 ± 2.66	t = 0.20, *p* = 0.838
Lunch	5.65 ± 2.21	5.68 ± 2.14	5.62 ± 2.28	t = 0.33, *p* = 0.742
Dinner	5.55 ± 2.42	5.78 ± 2.26	5.34 ± 2.55	t = 2.09, *p* = 0.037 *
**Snacking Behaviors** (Mean number of days in a week, 0–7)				
Morning Tea	4.31 ± 2.56	4.39 ± 2.64	4.24 ± 2.49	t = 0.71, *p* = 0.478
Afternoon Tea	3.60 ± 2.83	3.72 ± 2.80	3.48 ± 2.86	t = 0.97, *p* = 0.334
Supper	1.80 ± 2.48	1.79 ± 2.45	1.82 ± 2.50	t = −0.13, *p* = 0.900
**Physical Activity**				
Total Physical Activity score (1–5)	2.45 ± 0.61	2.42 ± 0.66	2.47 ± 0.55	t = −0.82, *p* = 0.412
**Physical Activity Level**				χ^2^ = 6.68, *p* = 0.035 *
Low (1.00–2.33)	236 (45.1)	123 (49.0)	113 (41.5)	
Moderate (2.34–3.66)	269 (51.4)	116 (46.2)	153 (56.3)	
High (3.67–5.00)	18 (3.4)	12 (4.8)	6 (2.2)	
**Body Composition**				
Weight (kg)	29.93 ± 10.57	30.10 ± 11.32	29.77 ± 9.85	t = 0.36, *p* = 0.717
Height (cm)	130.10 ± 9.74	130.13 ± 9.82	130.07 ± 9.69	t = 0.07, *p* = 0.943
BMI-for-age z score (BAZ)	0.15 ± 1.71	0.16 ± 1.76	0.14 ± 1.66	t = 0.09, *p* = 0.926
**Body Weight Status**				χ^2^ = 0.54, *p* = 0.909
Thinness (BAZ < −2SD)	41 (7.8)	18 (7.2)	23 (8.5)	
Normal (−2SD ≤ BAZ ≤ +1SD)	325 (62.1)	158 (62.9)	167 (61.4)	
Overweight (+1SD < BAZ ≤ +2SD)	70 (13.4)	32 (12.7)	38 (14.0)	
Obesity (BAZ > +2SD)	87 (16.6)	43 (17.1)	44 (16.2)	
**Cognitive Performance**				
Cognitive Performance score	89.33 ± 15.87	89.99 ± 15.33	88.73 ± 16.36	t = 0.91, *p* = 0.365
**Cognitive Performance Level**				χ^2^ = 3.43, *p* = 0.488
Low and borderline (<80)	118 (22.6)	50 (19.9)	68 (25.0)	
Below average (80–89)	146 (27.9)	69 (27.5)	77 (28.3)	
Average (90–109)	189 (36.1)	97 (38.6)	92 (33.8)	
High average (110–119)	42 (8.0)	23 (9.2)	19 (7.0)	
Superior (≥120)	28 (5.4)	12 (4.8)	16 (5.9)	

^1^ Comparison of pre-intervention characteristics between IG and CG was performed for continuous and categorical variables by using an independent sample t-test and chi-square tests. Significant differences between IG and CG were determined at *p*-value < 0.05. ^2^ Based on data from Department of Statistics Malaysia (2018), parental monthly income was categorized into B40 (<RM 3860), M40 (RM 3860—RM 8319), or T20 (>RM 8319) [33].

**Table 2:** Changes in eating behaviors, physical activity, BAZ, and cognitive performance from pre-intervention to post-intervention and at 3-month follow-up.

Variables	Changes from Pre- to Post-Intervention ^a^		Changes from Pre- to Post- Intervention II ^a^	
	Mean Change ± SD	t Value (*p*-Value)	Mean Change ± SD	t Value (*p*-Value)
**Eating Behavior**				
**Breakfast**				
IG (*n* = 251)	0.32 ± 3.48	1.44 (0.153)	0.55 ± 3.39	2.52 (0.012) *
CG (*n* = 272)	−0.60 ± 3.28	−3.02 (0.003) *	−0.63 ± 3.18	−3.14 (0.002) *
**Lunch**				
IG (*n* = 251)	0.25 ± 2.62	1.52 (0.130)	0.30 ± 2.44	1.90 (0.059)
CG (*n* = 272)	−1.26 ± 2.90	−7.15 (<0.001) *	−0.98 ± 2.85	−5.50 (<0.001) *
**Dinner**				
IG (*n* = 251)	−0.08 ± 2.95	−0.45 (0.654)	0.28 ± 2.52	1.73 (0.084)
CG (*n* = 272)	−0.82 ± 2.98	−4.52 (<0.001) *	−0.76 ± 2.81	−4.30 (<0.001) *
**Morning Tea**				
IG (*n* = 251)	0.26 ± 3.66	1.14 (0.256)	1.08 ± 2.96	5.68 (<0.001) *
CG (*n* = 272)	−0.50 ± 3.25	−2.54 (0.012) *	−1.06 ± 3.34	−2.03 (<0.001) *
**Afternoon Tea**				
IG (*n* = 251)	0.08 ± 3.70	0.32 (0.746)	−0.75 ± 3.46	−3.38 (0.001) *
CG (*n* = 272)	−0.52 ± 3.16	−2.72 (0.007) *	−0.36 ± 3.22	−1.78 (0.077)
**Supper**				
IG (*n* = 251)	−0.22 ± 2.95	−1.16 (0.249)	−0.36 ± 2.81	−2.02 (0.045) *
CG (*n* = 272)	−0.14 ± 2.79	−0.80 (0.423)	−0.15 ± 2.74	−0.90 (0.372)
**Physical activity score**				
IG (*n* = 251)	0.37 ± 0.83	7.12 (<0.001) *	0.18 ± 0.86	3.26 (0.001) *
CG (*n* = 272)	0.07 ± 0.69	1.75 (0.082)	0.02 ± 0.67	0.51 (0.609)
**Body weight**				
IG (*n* = 251)	1.15 ± 2.52	7.21 (<0.001) *	2.37 ± 2.99	12.29 (<0.001) *
CG (*n* = 272)	1.59 ± 2.41	10.86 (<0.001) *	3.11 ± 2.09	23.63 (<0.001) *
**Height**				
IG (*n* = 251)	1.95 ± 1.23	24.84 (<0.001) *	3.60 ± 1.80	31.15 (<0.001) *
CG (*n* = 272)	2.13 ± 1.73	20.37 (<0.001) *	3.66 ± 2.45	23.70 (<0.001) *
**BMI-for-age z score (BAZ)**				
IG (*n* = 251)	−0.03 ± 0.37	−1.06 (0.289)	−0.02 ± 0.49	−0.50 (0.615)
CG (*n* = 272)	0.08 ± 0.60	2.15 (0.033) *	0.20 ± 0.55	5.83 (<0.001) *
**Cognitive Performance**				
IG (*n* = 251)	13.12 ± 14.54	14.29 (<0.001) *	19.17 ± 16.42	18.15 (<0.001) *
CG (*n* = 272)	9.67 ± 14.06	11.35 (<0.001) *	14.78 ± 15.89	14.80 (<0.001) *

^a^ Differences in mean changes (post-intervention minus pre-intervention versus 3-month follow-up minus pre-intervention) within IG and CG were assessed using a paired-sample *t*-test. * Statistical significance was determined at *p* < 0.05.

**Table 3:** Changes in eating behaviors and physical activity, adjusted with covariates ^b^.

	**Breakfast**			**Lunch**			**Dinner**			**Morning Tea**		
	**Adjusted Beta (β)**	**95% CI**	***p*** **-Value**	**Adjusted Beta (β)**	**95% CI**	***p*** **-Value**	**Adjusted Beta (β)**	**95% CI**	***p*** **-Value**	**Adjusted Beta (β)**	**95% CI**	***p*** **-Value**
Intercept (SE)	4.41 (0.23)			5.37 (0.51)			4.17 (0.19)			3.98 (0.22)		
**Time**												
Pre-Intervention	Reference			Reference			Reference			Reference		
Post-I ^c^	−0.60	−1.00, −0.20	0.003 *	−1.26	−1.58, −0.93	<0.001 *	−0.82	−1.15, −0.48	<0.001 *	−0.50	−0.90, −0.10	0.015 *
Post-II ^d^	−0.71	−1.10, −0.33	<0.001 *	−1.05	−1.36, −0.73	<0.001 *	−0.86	−1.17, −0.54	<0.001 *	−1.06	−1.44, −0.69	<0.001 *
**Group**												
Comparison	Reference			Reference			Reference			Reference		
Intervention	−0.12	−0.61, 0.37	0.629	0.74	−0.24, 1.72	0.137	0.54	0.13, 0.95	0.010 *	0.04	−0.45, 0.53	0.882
**Interaction between time and group**												
Pre x Comparison	Reference			Reference			Reference			Reference		
Post-I x Intervention	1.20	0.57, 1.83	<0.001 *	1.28	0.77, 1.79	<0.001 *	0.50	−0.04, 1.04	0.068	0.84	0.20, 1.48	0.010 *
Post-II x Intervention	1.57	0.95, 2.18	<0.001 *	1.27	0.76, 1.77	<0.001 *	0.98	0.48, 1.47	<0.001 *	2.31	1.70, 2.91	<0.001 *
**Children’s age**												
Level 2 (10–11 years)	Reference			Reference			Reference			Reference		
Level 1 (7–9 years)	−0.17	−0.49, 0.14	0.277	0.02	−0.27, 0.31	0.887	0.18	−0.09, 0.45	0.196	0.25	−0.04, 0.54	0.095
**Sex**												
Girls	Reference			Reference			Reference			Reference		
Boys	0.11	−0.19, 0.41	0.474	−0.06	−0.33, 0.22	0.691	0.08	−0.18, 0.35	0.523	−0.12	−0.40, 0.16	0.406
**Father’s Education Level**												
Secondary	Reference			Reference			Reference			Reference		
Tertiary	0.58	0.03, 1.12	0.039 *	0.003	−0.49, 0.49	0.991	0.29	−0.18, 0.76	0.220	0.09	−0.42, 0.60	0.725
Primary or below	−0.25	−0.78, 0.27	0.343	0.01	−0.46, 0.49	0.950	−0.15	−0.60, 0.30	0.511	−0.25	−0.73, 0.24	0.323
**Mother’s Education Level**												
Secondary	Reference			Reference			Reference			Reference		
Tertiary	−0.67	−1.18, −0.16	0.011 *	−0.44	−0.90, 0.02	0.060	−0.20	−0.64, 0.24	0.367	−0.11	−0.59, 0.37	0.656
Primary or below	−0.10	−0.60, 0.41	0.708	0.03	−0.42, 0.49	0.880	−0.004	−0.44, 0.43	0.986	0.13	−0.34, 0.60	0.587
**School Type**												
SK	Reference			Reference			Reference			Reference		
SJKC	1.32	0.96, 1.68	<0.001 *	−0.16	−1.10, 0.78	0.739	2.19	1.88, 2.50	<0.001 *	−0.05	−0.39, 0.28	0.752
SJKT	0.66	0.25, 1.07	0.002 *	1.11	0.14, 2.08	0.025 *	1.52	1.17, 1.88	<0.001 *	0.96	0.58, 1.34	<0.001 *
	**Afternoon Tea**			**Supper**			**Physical Activity**					
	**Adjusted Beta (β)**	**95% CI**	***p*** **-Value**	**Adjusted Beta (β)**	**95% CI**	***p*** **-Value**	**Adjusted Beta (β)**	**95% CI**	***p*** **-Value**			
Intercept (SE)	3.67 (0.23)			1.17 (0.20)			2.39 (0.05)					
**Time**												
Pre-Intervention	Reference			Reference			Reference					
Post-I ^c^	−0.52	−0.92, −0.12	0.011 *	−0.14	−0.48, 0.20	0.433	0.07	−0.01, 0.16	0.098			
Post-II ^d^	−0.40	−0.81, −0.002	0.049 *	−0.14	−0.47, 0.19	0.411	0.03	−0.05, 0.12	0.460			
**Group**												
Comparison	Reference			Reference			Reference					
Intervention	0.28	−0.23, 0.80	0.282	−0.32	−0.77, 0.12	0.154	−0.07	−0.18, 0.04	0.185			
**Interaction between time and group**												
Pre x Comparison	Reference			Reference			Reference					
Post-I x Intervention	0.48	−0.16, 1.12	0.139	−0.01	−0.55, 0.53	0.969	0.34	0.20, 0.47	<0.001 *			
Post-II x Intervention	−0.30	−0.94, 0.34	0.355	−0.15	−0.69, 0.38	0.569	0.19	0.05, 0.33	0.008 *			
**Children’s age**												
Level 2 (10–11 years)	Reference			Reference			Reference					
Level 1 (7–9 years)	0.18	−0.14, 0.51	0.269	0.32	0.03, 0.60	0.030 *	−0.06	−0.14, 0.02	0.159			
**Sex**												
Girls	Reference			Reference			Reference					
Boys	−0.05	−0.37, 0.26	0.741	0.33	0.06, 0.60	0.018 *	0.14	0.07, 0.22	<0.001 *			
**Father’s Education Level**												
Secondary	Reference			Reference			Reference					
Tertiary	−0.46	−1.02, 0.11	0.112	−0.07	−0.56, 0.42	0.773	−0.06	−0.20, 0.08	0.422			
Primary or below	0.03	−0.51, 0.58	0.900	−0.24	−0.72, 0.23	0.310	−0.02	−0.16, 0.11	0.738			
**Mother’s Education Level**												
Secondary	Reference			Reference			Reference					
Tertiary	−0.01	−0.54, 0.52	0.967	0.01	−0.45, 0.48	0.950	−0.002	−0.13, 0.13	0.976			
Primary or below	0.008	−0.51, 0.53	0.976	0.14	−0.31, 0.59	0.543	0.01	−0.12, 0.14	0.888			
**School Type**												
SK	Reference			Reference			Reference					
SJKC	−1.15	−1.52, −0.78	<0.001 *	0.16	−0.16, 0.48	0.320	0.15	0.06, 0.24	0.001 *			
SJKT	0.79	0.37, 1.22	<0.001 *	1.37	1.00, 1.74	<0.001 *	0.04	−0.06, 0.14	0.448			

^b^ Covariates included children’s age, sex, parental education levels, and school type. ^c^ Post-I = Post-Intervention. ^d^ Post-II = 3-month follow-up. * Statistical significance was determined at *p* < 0.05.

**Table 4:** Changes in nutritional status and cognitive performance, adjusted with covariates ^b^.

	Body Weight			Height			BAZ			Cognitive Performance		
	Adjusted Beta (β)	95% CI	*p*-Value	Adjusted Beta (β)	95% CI	*p*-Value	Adjusted Beta (β)	95% CI	*p*-Value	Adjusted Beta (β)	95% CI	*p*-Value
Intercept (SE)	32.45 (0.97)			135.29 (0.87)			−0.26 (0.19)			87.35 (1.49)		
**Time**												
Pre-Intervention	Reference			Reference			Reference			Reference		
Post-I ^c^	1.59	1.34, 1.83	<0.001 *	2.13	1.96, 2.31	<0.001 *	0.08	0.02, 0.14	0.011 *	9.67	7.97, 11.37	<0.001 *
Post-II ^d^	3.00	2.76, 3.23	<0.001 *	3.55	3.29, 3.81	<0.001 *	0.19	0.13, 0.25	<0.001 *	13.71	11.79, 15.63	<0.001 *
**Group**												
Comparison	Reference			Reference			Reference			Reference		
Intervention	−0.10	−1.89, 1.70	0.916	0.07	−1.37, 1.50	0.926	−0.16	−0.48, 0.16	0.337	1.60	−1.15, 4.35	0.253
**Interaction between time and group**												
Pre x Comparison	Reference			Reference			Reference			Reference		
Post-I x Intervention	−0.19	−0.57, 0.19	0.333	−0.05	−0.34, 0.23	0.706	−0.08	−0.17, 0.02	0.108	3.98	1.28, 6.69	0.004 *
Post-II x Intervention	−0.47	−0.85, −0.09	0.016 *	0.27	−0.14, 0.68	0.202	−0.20	−0.30, −0.11	<0.001 *	4.35	1.29, 7.41	0.005 *
**Children’s age**												
Level 2 (10–11 years)	Reference			Reference			Reference			Reference		
Level 1 (7–9 years)	−6.37	−7.89, −4.85	<0.001 *	−12.08	−13.52, −10.63	<0.001 *	0.21	−0.10, 0.53	0.182	1.45	−0.91, 3.81	0.229
**Sex**												
Girls	Reference			Reference			Reference			Reference		
Boys	1.96	0.50, 3.42	0.009 *	−0.15	−1.53, 1.24	0.834	0.49	0.18, 0.79	0.002 *	1.13	−1.14, 3.39	0.330
**Father’s Education Level**												
Secondary	Reference			Reference			Reference			Reference		
Tertiary	−0.90	−3.53, 1.73	0.500	0.70	−1.80, 3.19	0.584	−0.38	−0.92, 0.16	0.172	2.20	−1.89, 6.29	0.292
Primary or below	1.96	−0.56, 4.48	0.127	1.49	−0.91, 3.89	0.223	0.18	−0.34, 0.70	0.507	−3.81	−7.74, 0.11	0.057
**Mother’s Education Level**												
Secondary	Reference			Reference			Reference			Reference		
Tertiary	2.00	−0.46, 4.47	0.111	1.56	−0.78, 3.90	0.192	0.41	−0.10, 0.92	0.113	0.80	−3.04, 4.63	0.683
Primary or below	−0.05	−2.46, 2.37	0.970	−0.36	−2.66, 1.93	0.755	0.17	−0.33, 0.66	0.515	−2.39	−6.15, 1.36	0.211
**School Type**												
SK	Reference			Reference			Reference			Reference		
SJKC	1.57	−0.15, 3.28	0.073	4.07	2.44, 5.70	<0.001 *	0.04	−0.31, 0.40	0.811	5.94	3.27, 8.61	<0.001 *
SJKT	−2.96	−4.94, −0.99	0.003 *	2.70	0.82, 4.58	0.005 *	−0.14	−0.55, −0.27	0.507	−8.00	−11.08, −4.93	<0.001 *

^b^ Covariates included children’s age, sex, parental education levels, and school type. ^c^ Post-I = Post-Intervention. ^d^ Post-II = 3-month follow-up. * Statistical significance was determined at *p* < 0.05.

## Data Availability

Data sharing is not applicable to this article.

## References

[1] World Health Organization (2006). News and Information: The WHO Global Database on BMI. Nutrition.

[2] Poh B.K., Ng B.K., Haslinda M.D.S., Shanita S.N., Wong J.E., Budin S.B., Ruzita A.T., Ng L.O., Khouw I., Norimah A.K. (2013). Nutritional status and dietary intakes of children aged 6 months to 12 years: Findings of the Nutrition Survey of Malaysian Children (SEANUTS Malaysia). Br. J. Nutr..

[3] Institute for Public Health (IPH) National Health and Morbidity Survey (NHMS) 2019: Vol. I: NCDs—Non-Communicable Diseases: Risk Factors and other Health Problems. http://www.iku.gov.my/images/IKU/Document/REPORT/NHMS2019/Report_NHMS2019-NCD_v2.pdf.

[4] Tee E.S., Nurliyana A.R., Karim N.A., Jan Mohamed H.J.B., Tan S.Y., Appukutty M., Hopkins S., Thielecke F., Ong M.K., Ning C. (2018). Breakfast consumption among Malaysian primary and secondary school children and relationship with body weight status—Findings from the MyBreakfast Study. Asia Pac. J. Clin. Nutr..

[5] Khambalia A.Z., Lim S.S., Gill T., Bulgiba A.M. (2012). Prevalence and sociodemographic factors of malnutrition among children in Malaysia. Food Nutr. Bull..

[6] Su T.T., Sim P.Y., Nahar A.M., Majid H.A., Murray L.J., Cantwell M.M., Al-Sadat N., Jalaludin M.Y. (2014). Association between self-reported physical activity and indicators of body composition in Malaysian adolescents. Prev. Med..

[7] Rezali F.W., Chin Y.S., Yusof M., Nisak B. (2012). Obesity-related behaviors of Malaysian adolescents: A sample from Kajang district of Selangor state. Nutr. Res. Pract..

[8] Freedman D.S., Mei Z., Srinivasan S.R., Berenson G.S., Dietz W.H. (2007). Cardiovascular risk factors and excess adiposity among overweight children and adolescents: The Bogalusa Heart Study. J. Pediatr..

[9] Whitlock E.P., Williams S.B., Gold R., Smith P.R., Shipman S.A. (2005). Screening and interventions for childhood overweight: A summary of evidence for the US Preventive Services Task Force. Pediatrics.

[10] Lazzeri G., Rossi F., Pammolli A., Pilato V., Pozzi T., Giacchi M. (2008). Underweight and overweight among children and adolescents in Tuscany (Italy). Prevalence and short-term trends. J. Prev. Med. Hyg..

[11] Luder E., Alton I. (2005). The Underweight Adolescent. Guidelines for Adolescent Nutritional Services.

[12] Poh B.K., Rojroonwasinkul N., Le Nyugen B.K., Budiman B., Ng L.O., Soonthorndhada K., Xuyen H.T., Deurenberg P., Parikh P. (2013). Relationship between anthropometric indicators and cognitive performance in Southeast Asian school-aged children. Br. J. Nutr..

[13] Hayashi C., Krasevec J., Kumapley R., Mehra V., de Onis M., Borghi E. (2017). Levels and Trends in Child Malnutrition. UNICEF/WHO/World Bank Group Joint Child Malnutrition Estimates: Key Findings of the 2017 Edition. https://www.who.int/nutgrowthdb/2018-jme-brochure.pdf.

[14] Institut of Public Health (IPH) National Health & Morbidity Survey (NHMS) 2017: Adolecent Nutrition Survey 2017, Malaysia; Institute for Public Health, National Institutes of Health, Ministry of Health, Selangor, Malaysia: 2017. http://iku.moh.gov.my/images/IKU/Document/REPORT/NHMS2017/ANS_KEDAH.pdf.

[15] Chong K.H., Wu S.K., Noor Hafizah Y., Bragt M.C., Poh B.K. (2016). SEANUTS Malaysia Study Group. Eating habits of Malaysian children: Findings of the South East Asian Nutrition Surveys (SEANUTS). Asia-Pac. J. Public Health.

[16] Lee S.T., Wong J.E., Shanita S.N., Ismail M.N., Deurenberg P., Poh B.K. (2014). Daily physical activity and screen time, but not other sedentary activities, are associated with measures of obesity during childhood. Int. J. Environ. Res. Publ. Health.

[17] Chin Y.S., Mohd Nasir M. (2009). Eating behaviors among female adolescents in Kuantan district, Pahang, Malaysia. Pak. J. Nutr..

[18] Lee P.Y., Cheah W.I., Chang C.T., Raudzah S. (2012). Childhood obesity, self-esteem and health-related quality of life among urban primary schools children in Kuching, Sarawak, Malaysia. Malays. J. Nutr..

[19] Nasir M.T., Norimah A.K., Hazizi A.S., Nurliyana A.R., Loh S.H., Suraya I. (2012). Child feeding practices, food habits, anthropometric indicators and cognitive performance among preschoolers in Peninsular Malaysia. Appetite.

[20] Mensink F., Schwinghammer S.A., Smeets A. (2012). The Healthy School Canteen programme: A promising intervention to make the school food environment healthier. J. Environ. Public Health..

[21] Shariff Z.M., Bukhari S.S., Othman N., Hashim N., Ismail M., Jamil Z., Kasim S.M., Paim L., Samah B.A., Hussein Z.A.M. (2008). Nutrition education intervention improves nutrition knowledge, attitude and practices of primary school children: A pilot study. Int. Electron. J. Health Educ..

[22] Fox M.K., Dodd A.H., Wilson A., Gleason P.M. (2009). Association between school food environment and practices and body mass index of US public school children. J. Am. Diet. Assoc..

[23] World Health Organization (WHO) Mental Health: Depression, Lets Talk. https://www.who.int/mental_health/management/depression/en/2017.

[24] Ministry of Eduaction Malaysia (MOE) Education System Chart 2018. www.moe.gov.my/index.php/en/arkib/sistem-pendidikan.

[25] Lee A., Cheng F.F.K., Fung Y., St Leger L. (2006). Can Health Promoting Schools contribute to the better health and wellbeing of young people? The Hong Kong experience. J. Epidemiol. Comunity Health.

[26] Tanaka N., Miyoshi M. (2012). School lunch program for health promotion among children in Japan. Asia Pac. J. Clin. Nutr..

[27] Meyer P.A., Yoon P.W., Kaufmann R.B. (2013). Introduction: CDC Health Disparities and Inequalities Report-United States, 2013. MMWR Suppl..

[28] Ruth C.S., Suriani I., Norliza A., Lim P.Y., Ibrahim A.N. (2020). Systematic review: Effect of health education intervention on improving knowledge, attitudes and practices of adolescents of malnutrition. Nutrients.

[29] Ruzita A.T., Wan Azdie M.A., Ismail M.N. (2007). The effectiveness of nutrition education programme for primary school children. Malays. J. Nutr..

[30] Chin Y.S., Tee E.S., Zawiah H., Rasyedah A.R., Norimah A.K. (2020). Effectiveness of a nutrition education intervention for primary school children: The Healthy Kids Programme, Malaysia. Mal. J. Med. Health Sci..

[31] Teo C.H., Chin Y.S., Lim P.Y., Shahril Azian H.M., Zalilah M.S. (2019). School-based intervention that integrates nutrition education and supportive healthy school food environment among Malaysian primary school children: A study protocol. BMC Public Health.

[32] Miyoshi M., Tsuboyama-Kasaoka N., Nishi N. (2012). School-based „Shokuiku“ program in Japan: Application to nutrition education in Asian countries. Asia Pac. J. Clin. Nutr..

[33] Aday L.A., Cornelius L.J. (2006). Designing and Conducting Health Surveys: A Comprehensive Guide.

[34] Yusop N.B., Shariff Z.M., Hwu T.T., Talib R.A., Spurrier N. (2018). The effectiveness of a stage-based lifestyle modification intervention for obese children. BMC Public Health.

[35] World Health Organization (WHO) Health in 2015: From MDGs, Millennium Development Goals to SDGs, Sustainable Development Goals. https://www.who.int/gho/publications/mdgs-sdgs/en/.

[36] Bandura A. (2001). Social cognitive theory: An agentic perspective. Annu. Rev. Psychol..

[37] Ministry of Education Malaysia (MOE) (2011). Panduan Pengurusan Kantin Sekolah Sihat. https://ecepakk3243tk.files.wordpress.com/2018/02/panduan-hidangan-sihat-di-kantin-sekolah.pdf.

[38] Department of Statistics Malaysia (2018). Demographic Statistics Third Quarter (Q3) 2018 from Malaysia. https://www.dosm.gov.my/v1/index.php?r=column/cthemeByCat&cat=430&bul_id=bGs2eUViWlNoTDQybFJwanlEQW9YZz09&menu_id=L0pheU43NWJwRWVSZklWdzQ4TlhUUT09.

[39] Aggio D., Fairclough S., Knowles Z., Graves L. (2016). Validity and reliability of a modified english version of the physical activity questionnaire for adolescents. Arch. Public Health.

[40] Kowalski K.C., Crocker P.R., Donen R.M. (2004). The physical activity questionnaire for older children (PAQ-C) and adolescents (PAQ-A) manual. Coll. Kinesiol. Univ. Sask..

[41] Dan S.P., Mohd Nasir M.T., Zalilah M.S. (2007). Sex and ethnic differentials in physical activity levels of adolescents in Kuantan. Malays. J. Nutr..

[42] World Health Organization (WHO) (2009). WHO AnthroPlus for Personal Computers Manual: Software for Assessing Growth of the World‘s Children and Adolescents. https://www.who.int/growthref/tools/en/.

[43] AnthroPlus WHO. http://who-anthroplus.freedownloadscenter.com/windows/.

[44] Raven J. (2008). Raven’s e Educational: Coloured Progressive Matrices (CPM).

[45] Gupta S.K. (2011). Intention-to-treat concept: A review. Perpect. Clin. Res..

[46] Rampersaud G.C. (2009). Benefits of breakfast for children and adolescents: Update and recommendations for practitioners. Am. J. Lifestyle Med..

[47] Shah P., Misra A., Gupta N., Hazra D.K., Gupta R., Seth P., Agarwal A., Gupta A.K., Jain A., Kulshreshta A. (2010). Improvement in nutrition-related knowledge and behaviour of urban Asian Indian school children: Findings from the ‘Medical education for children/Adolescents for Realistic prevention of obesity and diabetes and for healthy aGeing’ (MARG) intervention study. Br. J. Nutr..

[48] Sharif Ishak S., Chin Y.S., Mohd Taib M.N., Chan Y.M., Shariff Z.M. (2020). Effectiveness of a school-based intervention on knowledge, attitude and practice on healthy lifestyle and body composition in Malaysian adolescents. BMC Pediatrics.

[49] Asakura K., Todoriki H., Sasaki S. (2017). Relationship between nutrition knowledge and dietary intake among primary school children in Japan: Combined effect of children’s and their guardians’ knowledge. J. Epidemiol..

[50] Grosso G., Mistretta A., Turconi G., Cena H., Roggi C., Galvano F. (2013). Nutrition knowledge and other determinants of food intake and lifestyle habits in children and young adolescents living in a rural area of Sicily, South Italy. Public Health Nutr..

[51] Guthrie J.F., Derby B.M., Levy A.S. (1999). What People Know and Do not Know about Nutrition. America’s Eating Habits: Changes and Consequences. https://www.ers.usda.gov/webdocs/publications/42215/5842_aib750m_1_.pdf?v=0.

[52] Durlak J.A., Weissberg R.P., Dymnicki A.B., Taylor R.D., Schellinger K.B. (2011). The impact of enhancing students’ social and emotional learning: A meta-analysis of school-based universal interventions. Child. Dev..

[53] Singhal N., Misra A., Shah P., Gulati S. (2010). Effects of controlled school-based multi-component model of nutrition and lifestyle interventions on behavior modification, anthropometry and metabolic risk profile of urban Asian Indian adolescents in North India. Eur. J. Clin. Nutr..

[54] Mary E.S., D‘souza A., Roach E.J. (2014). Effectiveness of a lifestyle management program on knowledge and lifestyle practices among adolescents. Nitte Univ. J. Health Sci..

[55] Terry-McElrath Y.M., O’malley P.M., Delva J., Johnston L.D. (2009). The school food environment and student body mass index and food consumption: 2004 to 2007 national data. J. Adolesc Health..

[56] Shepherd J., Harden A., Rees R., Brunton G., Garcia J., Oliver S., Oakley A. (2005). Young people and healthy eating: A systematic review of research on barriers and facilitators. Heal. Educ. Res..

[57] Bevans K.B., Sanchez B., Teneralli R., Forrest C.B. (2011). Children‘s eating behavior: The importance of nutrition standards for foods in schools. J. Sch. Health.

[58] Reilly K.L., Nathan N., Wiggers J., Yoong S.L., Wolfenden L. (2018). Scale up of a multi-strategic intervention to increase implementation of a school healthy canteen policy: Findings of an intervention trial. BMC Public Health.

[59] Andrade S., Lachat C., Aviles A.M.O., Verstraeten R., Huybregts L., Roberfroid D., Andrade D., Van Camp J., Rojas R., Donoso S. (2014). A school-based intervention improves physical fitness in Ecuadorian adolescents: A cluster-randomized controlled trial. Int. J. Behav. Nutr. Phys. Act..

[60] Sutherland R., Campbell E., Lubans D.R., Morgan P.J., Okely A.D., Nathan N., Wolfenden L., Wiese J., Gillham K., Hollis J. (2015). ‘Physical Activity 4 Everyone’ school-based intervention to prevent decline in adolescent physical activity levels: 12 month (mid-intervention) report on a cluster randomised trial. Br. J. Sports Med..

[61] Koo H.C., Poh B.K., Abd Talib R. (2018). The GReat-Child™ Trial: A Quasi-Experimental Intervention on Whole Grains with Healthy Balanced Diet to Manage Childhood Obesity in Kuala Lumpur, Malaysia. Nutrients.

[62] Hansen K., Joshi H. (2008). Millennium Cohort Study Third Survey: A User’s Guide to Initial Findings: Centre for Longitudinal Studies.

[63] Pieper J.R., Whaley S.E. (2011). Healthy eating behaviors and the cognitive environment are positively associated in low-income households with young children. Appetite.

[64] Hoyland A., Dye L., Lawton C.L. (2009). A systematic review of the effect of breakfast on the cognitive performance of children and adolescents. Nutr. Res. Rev..

[65] Li Y., Dai Q., Jackson J.C., Zhang J. (2008). Overweight is associated with decreased cognitive functioning among school-age children and adolescents. Obesity.

[66] Best C., Neufingerl N., Van Geel L., van den Briel T., Osendarp S. (2010). The nutritional status of school-aged children: Why should we care?. Food Nutr. Bull..

[67] Koo H.C., Jalil S.N.A., Ruzita A.T. (2015). Breakfast eating pattern and ready-to-eat cereals consumption among schoolchildren in Kuala Lumpur. Malays. J. Med. Sci..

[68] Teng C.Y., Chin Y.S., Taib M.N.M., Chan Y.M. (2018). Evaluation of the Effectiveness of a 3-Year, Teacher-Led Healthy Lifestyle Program on Eating Behaviors Among Adolescents Living in Day School Hostels in Malaysia. Food Nutr. Bull..

[69] Gleason P.M., Dodd A.H. (2009). School breakfast program but not school lunch program participation is associated with lower body mass index. J. Am. Diet. Assoc..

[70] Nurul-Fadhilah A., Teo P.S., Huybrechts I., Foo L.H. (2013). Infrequent breakfast consumption is associated with higher body adiposity and abdominal obesity in Malaysian school-aged adolescents. PLoS ONE.

[71] El Harake M.D., Kharroubi S., Hamadeh S.K., Jomaa L. (2018). Impact of a Pilot School-Based Nutrition Intervention on Dietary Knowledge, Attitudes, Behavior and Nutritional Status of Syrian Refugee Children in the Bekaa, Lebanon. Nutrients.

[72] Waters E., De Silva-Sanigorski A., Burford B.J., Brown T., Campbell K.J., Gao Y., Armstrong R., Prosser L., Summerbell C.D. (2014). Interventions for preventing obesity in children. Sao Paulo Med. J..

[73] Chung K.H., Chiou H.Y., Chen Y.H. (2015). Psychological and physiological correlates of childhood obesity in Taiwan. Sci. Rep..

[74] Kristjansson B., Petticrew M., Macdonald B., Krasevec J., Janzen L., Greenhalgh T., Wells G.A., MacGowan J., Farmer A.P., Shea B. (2007). School feeding for improving the physical and psychosocial health of disadvantaged students. Cochrane Database Syst. Rev..

